# Comparison of prehospital tidal volume delivery performance between automated transport ventilators and bag-valve devices in out-of-hospital cardiac arrest patients: does the pressure limit matter?

**DOI:** 10.3389/fmed.2026.1826906

**Published:** 2026-05-25

**Authors:** Chi-Hsin Chen, Ping-Hsun Yu, Cheng-Yi Fan, Chih-Hung Wang, Jiun-Wei Chen, Edward Pei-Chuan Huang

**Affiliations:** 1 Department of Emergency Medicine, National Taiwan University Hospital Hsin-Chu Branch, Hsinchu, Taiwan; 2 Department of Emergency Medicine, College of Medicine, National Taiwan University, Taipei, Taiwan; 3 Department of Life Science, School of Life Science, College of Science, National Taiwan Normal University, Taipei, Taiwan; 4 Emergency Department, Taipei Hospital, Ministry of Health and Welfare, New Taipei, Taiwan; 5 Institute of Molecular Medicine, National Tsing Hua University, Hsinchu, Taiwan; 6 Department of Emergency Medicine, National Taiwan University Hospital, Taipei, Taiwan; 7 Department of Emergency Medicine, National Taiwan University Hospital Yun-Lin Branch, Douliu, Taiwan

**Keywords:** automatic transport ventilator, outcome, out-of-hospital cardiac arrest, tidal volume, ventilation

## Abstract

**Background:**

Automatic transport ventilators (ATVs) are a potential option for ventilating patients experiencing out-of-hospital cardiac arrest (OHCA); however, their tidal volume delivery performance at different pressure limits compared with that of manual ventilation remains unclear.

**Methods:**

This retrospective study with quasi-experimental before-after features included adult patients with nontraumatic OHCA with prehospital advanced airway in Hsinchu county, Taiwan, in 2023. Participants were categorized into three groups: Group 1 (ventilated by ATVs; pressure limit: 45 cmH_2_O), Group 2 (ventilated by ATVs; pressure limit: 60 cmH_2_O), and Group 3 [manual ventilation by bag-valve devices (BVDs)]. The inspiratory tidal volume measured using Zoll^®^ X-series monitors after advanced airway placement in each group was compared using a linear mixed-effect model (LMM). The association between ventilation settings, delivered inspiratory tidal volume, and return of spontaneous circulation (ROSC) was investigated using logistic regression.

**Results:**

Groups 1, 2, and 3 comprised 41 (23.0%), 80 (44.9%), and 57 (32.0%) patients with OHCA, respectively. In the *post-hoc* LMM analysis, Group 1 had a significantly higher tidal volume than Group 2 (adjusted difference: 66.84 mL, 95% confidence interval: 21.27–112.42, *p* = 0.012). However, no significant differences were found between Groups 1 and 3 or between Groups 2 and 3. No significant association was observed between different ventilation settings or delivered inspiratory tidal volume and prehospital ROSC or any ROSC following resuscitation.

**Conclusion:**

ATVs demonstrated comparable delivered inspiratory tidal volume to BVD ventilation in this exploratory retrospective cohort. Increasing the pressure limit of ATVs from 45 to 60 cmH2O did not increase delivered inspiratory tidal volume. The optimal pressure settings of ATVs, formal feasibility and implementation outcomes, and their effects on clinical outcomes remain unclear and require prospective validation.

## Introduction

During the resuscitation process of patients with OHCA, providing essential perfusion and oxygen supply is crucial for restoring spontaneous circulation and improving outcomes (1). Therefore, both high-quality chest compression and effective ventilation are important tasks in advanced cardiac life support (ACLS), not only during in-hospital resuscitation but also in prehospital settings.

Although effective ventilation is also crucial in the resuscitation of patients with OHCA, the efficacy of ventilation during cardiopulmonary resuscitation (CPR) has not been as comprehensively investigated as the quality of chest compressions (2, 3). Current ACLS guidelines recommend maintaining a ventilation rate of one breath every 6 s after the establishment of an advanced airway to prevent hyperventilation (4). Both excessive and insufficient ventilation may be detrimental during resuscitation: excessive ventilation may increase intrathoracic pressure and impair venous return, whereas insufficient ventilation may lead to inadequate oxygen delivery and carbon dioxide clearance (5, 6). Mechanical ventilation or automatic transport ventilators (ATVs) may help provide a more consistent ventilation rate and reduce variability in delivered breaths compared with manual ventilation using bag-valve devices (BVDs) (3). Furthermore, such ATVs can decrease the manpower required for ventilation, which may be beneficial in prehospital settings. Despite the many potential advantages of ATVs, their ventilation efficacy has not been well investigated. Orlob et al. assessed the ventilation efficacy of an ATV in a cadaver model under continuous chest compressions. With the pressure limit set at 60 cmH2O, the median tidal volume was 274.8 mL, which was lower than the target tidal volume of 6 mL/kg of body weight (7). However, although each breath should deliver a tidal volume of 500–600 mL or 6–8 mL/kg of body weight to achieve adequate ventilation according to the guidelines, several studies have raised concerns about insufficient tidal volume delivered during active CPR because intrathoracic pressure may increase and respiratory compliance can vary under chest compression (3, 7, 8). On the other hand, delivering excessively high tidal volumes and hyperventilation can adversely affect blood flow and overall resuscitation outcomes (9). Therefore, the tidal volume delivery performance of ATVs and manual ventilation using BVDs should be compared. Furthermore, the commonly set inspiratory pressure limit to avoid pressure-induced injury during mechanical ventilation may also affect the actual delivered tidal volume during ongoing chest compression (3). Whether adjusting the preset pressure limit affects the tidal volume delivered during chest compressions requires investigation.

This study compared tidal volume delivery performance between BVDs and ATVs under different pressure limits and investigated the association between different ventilation settings and patient outcomes.

## Materials and methods

### Study design and setting

This retrospective, quasi-experimental, before-after study was conducted in Hsinchu county, Taiwan, between January 1, 2023, and December 31, 2023. Hsinchu County has a population of approximately 600,000 as of 2023, served by an emergency medical service (EMS) system comprising 20 branches and 399 emergency medical technicians (EMTs), including 39 paramedics. In this EMS system, mechanical CPR using the LUCAS device is the standard approach during transport unless the device cannot be applied because of patient-related factors, most commonly body habitus or size limitation. Overall, mechanical CPR was used in more than 90% of transported OHCA patients in this EMS system. The Institutional Review Board of National Taiwan University Hospital (No. 202401198RIND) approved this study, and the requirement for informed consent was waived due to the retrospective nature. The study was conducted in concordance with the Declaration of Helsinki.

### Participant selection

This study adopted the following inclusion and exclusion criteria. The total cohort included all EMS-attended patients with OHCA in Hsinchu County, Taiwan, in 2023. The exclusion criteria were as follows: (1) No resuscitation attempted (included refrained from resuscitation due to a do-not-resuscitation request, obviously deceased, and signs of spontaneous circulation upon arrival); (2) pregnant women; (3) traumatic arrests; (4) age younger than 18 years old; (5) no prehospital advanced airway was placed [endotracheal tube (ETT) or laryngeal mask airway (LMA)]; and (6) ventilation feedback monitoring was not applied. The criteria for obviously deceased patients included advanced decomposition, rigor mortis, charring of the body, decapitation, evisceration, and truncal dismemberment. In our EMS system, once resuscitation is initiated, prehospital termination of the effort is not permitted.

### Measurements

#### Ventilation methods and tidal volume delivery assessment

All included patients were ventilated either manually using a standard BVD (Ambu bag) for adults or automatically using a MicroVENT^®^ CPR Resuscitator. The MicroVENT^®^ CPR Resuscitator is an oxygen-powered automatic time-cycled ventilation device that provides unsynchronized breaths under a fixed rate of 10 breaths/min and an inspiratory to expiratory (I: E) ratio of 1:5. The preset tidal volume was between 500 and 600 mL, and no positive end-expiratory pressure was applied. Furthermore, the device features a pressure relief valve to prevent pressure-induced barotrauma. The comparison between ATV pressure limits had quasi-experimental before-after features because the pressure-limit setting was implemented sequentially over time. In our study, we set the limit at the preset limit of 45 cmH2O from January 1, 2023, to April 30, 2023, and adjusted the pressure limit to 60 cmH2O from May 1, 2023, to the end of the study period. On the other hand, BVD was used for ventilation if ATV was not used or equipped throughout the study period. The choice between ATV and BVD ventilation was not randomized. ATV ventilation was used when the responding EMS team was equipped with the ATV device and applied it after successful advanced airway placement, whereas BVD ventilation was used when ATV was not available, not equipped, or not applied by the responding team. There were no additional patient-level exclusion criteria specifically used to assign patients to ATV or BVD ventilation beyond the general study inclusion and exclusion criteria. Finally, the included patients were divided into three ventilation setting groups—Group 1: ventilated using ATVs, with the pressure limit set to 45 cmH2O; Group 2: ventilated using ATVs, with the pressure limit set to 60 cmH2O; and Group 3: ventilated manually using BVDs unsynchronized with chest compression by EMTs, with the pop-off valve closed according to ACLS guidelines (4).

After the establishment of an advanced airway, the Zoll X Series^®^ Real BVM help system was applied to provide real-time ventilation feedback as soon as possible. This device measures inspiratory flow at the patient airway, and the inspiratory tidal volume is calculated by integrating the inspiratory flow signal during each delivered breath. Therefore, the tidal volume reported in this study was interpreted as device-derived delivered inspiratory tidal volume, not directly measured expiratory tidal volume, alveolar ventilation, or comprehensive ventilation effectiveness during CPR (10). Furthermore, we retrospectively collected the measured tidal volume per 6 s until the patient arrived at the hospital or a maximum recording time of 20 min. All EMTs involved in this study were trained to attach ATVs and ventilation feedback devices following a standard protocol.

#### Additional variables

Additional variables were recorded based on Utstein reporting guidelines for OHCA and were collected from routinely maintained Hsinchu County EMS prehospital care records produced by EMTs after each OHCA event (11). Ventilation parameters were retrospectively extracted from Zoll X Series® ventilation feedback recordings when ventilation feedback monitoring was applied, and hospital outcomes were obtained from hospital medical records. Variables included basic patient information, such as age, sex, precomorbidities, witnessed arrests, bystander CPR, prehospital resuscitation management including administration of epinephrine and automatic external defibrillator defibrillation, and the type of advanced airway used (ETT or LMA).

### Outcomes

The outcome variables included were prehospital return of spontaneous circulation (ROSC), any ROSC during resuscitation in the emergency department, survival to discharge, and good functional outcome at discharge (defined as Cerebral Performance Categories scores 1 and 2).

### Statistical analysis

Continuous variables are presented as medians and interquartile ranges and were compared using the Mann–Whitney U test. Categorical and dichotomous variables are presented as absolute numbers (percentages) and were compared using Pearson’s chi-square test or Fisher’s exact test. The median and maximum tidal volumes at 1-min intervals for patients in the different ventilation setting groups are presented using a line chart. Delivered inspiratory tidal volume was modeled as the dependent variable, and ventilation group was included as the main fixed effect. Covariates, including age, sex, pre-existing comorbidities, witnessed arrest, bystander CPR, epinephrine administration, defibrillation, advanced airway type, and measurement time point, were included as fixed effects. Measurement time point represented the elapsed time from the start of ventilation feedback monitoring and was modeled as a continuous variable. Patient identifier was included as a random intercept to account for within-patient correlation among repeated measurements. A random slope for time was not included because the primary objective was to estimate the average between-group difference in delivered inspiratory tidal volume, and the available sample size was limited for more complex random-effect structures. Model convergence was checked, and model assumptions were assessed by visual inspection of residual plots. To compare the tidal volume between each pair of groups, *post hoc* pairwise comparisons were performed using the Bonferroni correction to adjust for multiple comparisons. Finally, the association between different ventilation settings, tidal volume measured, and patient outcomes was investigated using multivariable logistic regression. Two-tailed *p*-values < 0.05 were used to denote statistical significance and were adjusted to <0.017 after Bonferroni correction for comparisons between the three groups in the *post hoc* analysis. Statistical Package for the Social Sciences (IBM SPSS, version 26.0; IBM Corp., Armonk, NY, United States) was used for all statistical analyses.

## Results

### Characteristics of the participants

**Figure 1:**
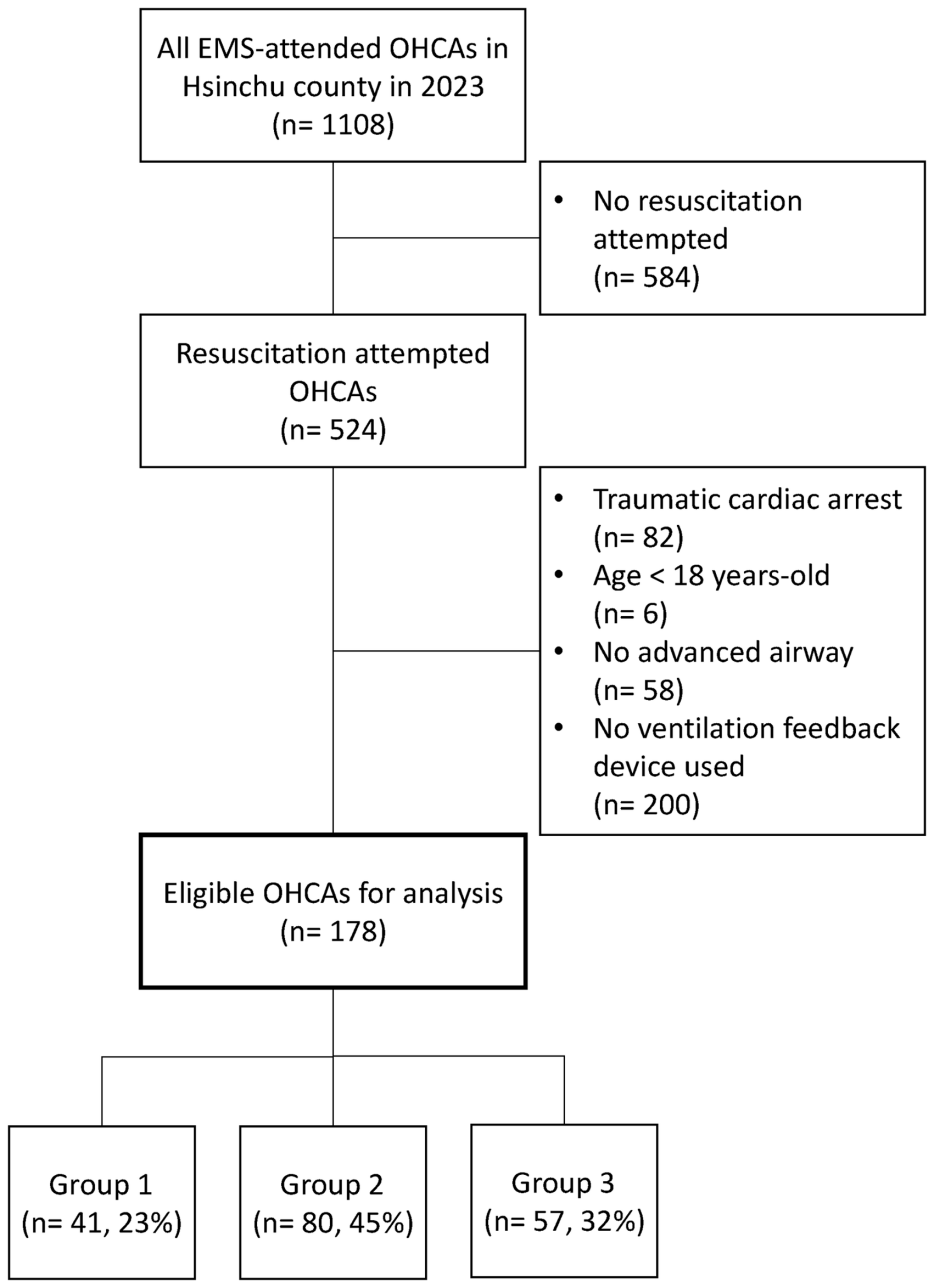
Flow diagram of included patients. Group 1: ventilated by ATVs with pressure limit set at 45 cm H_2_O. Group 2: ventilated by ATVs with pressure limit set at 60 cm H_2_O. Group 3: ventilated manually by BVDs. EMS, emergency medical service; OHCA, out-of-hospital cardiac arrest.

Figure 1 presents a flow diagram of the participant selection process. Among the 524 patients with OHCA with resuscitation attempted by EMS, we further excluded 82 patients with traumatic etiology, six patients under the age of 18 years, 58 patients without prehospital advanced airways, and 200 patients without ventilation feedback devices used. Ultimately, 178 patients were included in the final analysis. Among them, Group 1 included 41 (23.0%) patients, Group 2 included 80 (44.9%) patients, and Group 3 included 57 (32.0%) patients.

Table 1 presents the demographic characteristics, prehospital resuscitation information, tidal volume delivery performance, and outcomes of the included patients. The median age of the included patients was 68.5 years, and males accounted for 64.6%. For the advanced airway type, 62 (35.0%) patients received LMA insertion and 115 (65.0%) received ETT insertion. Prehospital ROSC and any ROSC were achieved in 14.6 and 26.4% of all included patients, respectively. Only two patients survived to discharge, and one patient had good functional outcomes upon discharge. No significant differences in demographic characteristics, prehospital resuscitation information, and outcomes were observed between the different ventilation setting groups.

**Table 1:** Comparison of demographics between patients ventilated by automatic transport ventilators with different pressure limits and by bag-valve devices.

Variable	Total (*n* = 178)	Group 1 (*n* = 41)	Group 2 (*n* = 80)	Group 3 (*n* = 57)	*p*
Age	68.5 (25.0)	68.0 (23.0)	71.0 (25.0)	66.0 (29.0)	0.795
Male	115 (64.6)	24 (58.5)	58 (72.5)	33 (57.9)	0.138
Precomorbidities					
DM	57 (32.0)	8 (19.5)	30 (37.5)	19 (33.3)	0.129
HTN	68 (38.2)	14 (34.1)	32 (40.0)	22 (38.6)	0.819
COPD/Asthma	6 (3.4)	1 (2.4)	3 (3.8)	2 (3.5)	0.929
CVA	10 (5.6)	3 (7.3)	4 (5.1)	3 (5.3)	0.869
Witness	71 (39.9)	14 (34.1)	31 (38.8)	26 (45.6)	0.500
Bystander CPR	150 (84.7)	34 (82.9)	66 (83.5)	50 (87.7)	0.747
Epinephrine	141 (79.2)	37 (90.2)	61 (76.3)	43 (75.4)	0.139
Defibrillation	55 (30.9)	10 (24.4)	28 (35.0)	17 (29.8)	0.478
Advanced airway					0.068
LMA	62 (35.0)	9 (22.0)	34 (43.0)	19 (33.3)	
ETT	115 (65.0)	32 (78.0)	45 (57.0)	38 (66.7)	
Tidal volume delivery performance					
Median delivered inspiratory tidal volume (mL)	333.0 (229.0)	403.0 (127.0)	275.0 (244.8)	363.0 (228.8)	0.016
Maximum delivered inspiratory tidal volume (mL)	577.5 (225.0)	534.0 (163.0)	567.5 (238.0)	644.0 (246.0)	0.024
Percentage of volume between 500 and 600 ml (%)	3.5 (16.0)	3.1 (20.1)	2.0 (12.0)	5.7 (16.0)	0.372
Outcome					
Prehospital ROSC	26 (14.6)	8 (19.5)	8 (10.0)	10 (17.5)	0.280
ROSC	47 (26.4)	12 (29.3)	18 (22.5)	17 (29.8)	0.564
Survival to discharge	2 (1.1)	1 (2.4)	1 (1.3)	0 (0.0)	0.523
Good functional outcome	1 (0.6)	1 (2.4)	0 (0.0)	0 (0.0)	0.186

Group 1: ventilated by ATVs with pressure limit set at 45 cmH2O.

Group 2: ventilated by ATVs with pressure limit set at 60 cmH2O.

Group 3: ventilated manually by Ambu bags.

COPD, chronic obstructive pulmonary disease; CPR, cardiopulmonary resuscitation; CVA, cerebral vascular disease; DM, diabetes mellitus; ETT, endotracheal tube; HTN, hypertension; LMA, laryngeal mask airway; ROSC, return of spontaneous circulation.

### Comparison of delivered inspiratory tidal volume under different ventilation settings

Table 1 presents the median delivered inspiratory tidal volume, maximum delivered inspiratory tidal volume, and percentage of delivered inspiratory tidal volume between 500 and 600 mL between the three ventilation setting groups. The median delivered inspiratory tidal volume was highest in Group 1, followed by those in Groups 3 and 2 (403.0, 363.0, and 275.0 mL, respectively; *p* = 0.016). The maximum delivered inspiratory tidal volume was highest in Group 3, followed by those in Groups 2 and 1 (644.0, 567.5, and 534.0 mL, respectively; *p* = 0.024). No significant difference in the percentage of volume between 500 and 600 mL was observed between the different ventilation setting groups. Subgroup analysis of patients receiving LMA or ETT insertion yielded similar results, with higher median tidal volume measured in the LMA group than ETT (Supplementary Table 1).

**Figure 2:**
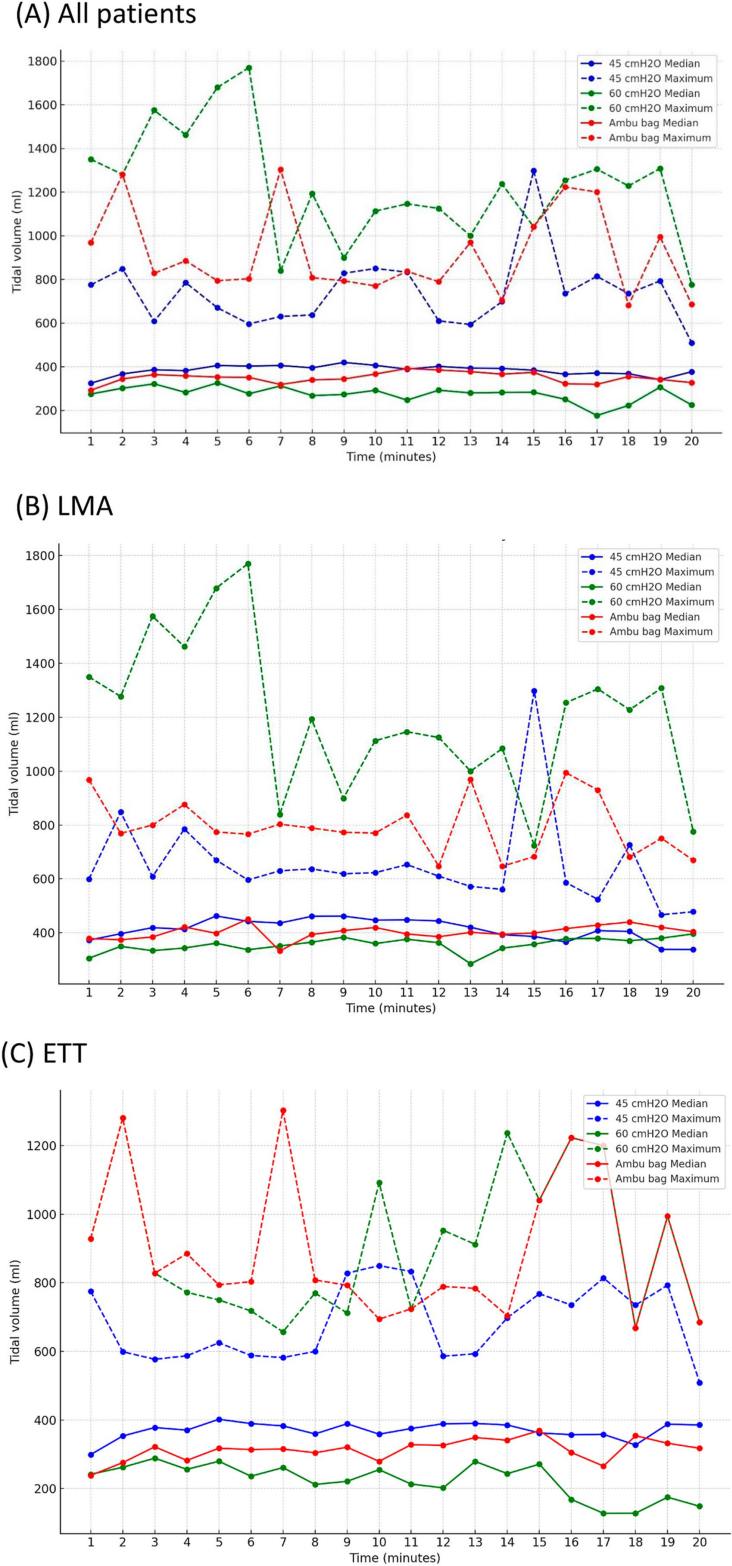
Median and maximum delivered inspiratory tidal volume in a 1-min interval between 3 ventilation setting groups and under different advanced airway managements: **(A)** All included patients; **(B)** Patients receiving LMA insertion; **(C)** Patients receiving ETT insertion. ETT, endotracheal tube; LMA, laryngeal mask.

Figure 2 presents the median and maximum delivered inspiratory tidal volumes at 1-min intervals between the three ventilation setting groups and under different advanced airway managements. In all included patients, the median delivered inspiratory tidal volume was highest in Group 1, followed by those in Groups 2 and 3 (Figure 2A). Despite these slight differences, the delivered inspiratory tidal volumes measured using the three ventilation methods were all approximately within the range of 300–400 mL. The maximum delivered inspiratory tidal volume within each minute exhibited greater variability, with the overall maximum delivered inspiratory tidal volumes measured in Group 1 being the lowest. Subgroup analysis using LMA and ETT revealed a similar trend to that observed in the overall patient population (Figures 2B,C).

Table 2 presents a comparison of delivered inspiratory tidal volume between the three ventilation setting groups using the LMM and adjusted for potential confounders. In the *post hoc* comparison, the delivered inspiratory tidal volume measured in Group 1 was higher than that measured in Group 2 (adjusted absolute difference: 66.84 mL, 95% confidence interval [CI]: 21.27–112.42; *p* = 0.012). No significant difference in delivered inspiratory tidal volume was observed between Groups 1 and 3 (adjusted absolute difference: 19.29 mL, 95% CI: −27.47 to 66.05; *p* = 1.000). Similarly, no significant difference was observed between Groups 2 and 3 (adjusted absolute difference: −47.56 mL, 95% CI: −88.08 to −7.04; *p* = 0.064). For the advanced airway type, patients who underwent ETT insertion had slightly lower delivered inspiratory tidal volumes than those who underwent LMA insertion (adjusted absolute difference: −57.30 mL, 95% CI: −98.20 to −16.39; *p* = 0.006). In additional stratified analyses by advanced airway type, the direction of the between-group differences was generally consistent with the overall analysis in both airway strata, but no pairwise comparison remained statistically significant after Bonferroni correction (Supplementary Table 2). No significant interaction was observed between ventilation group and advanced airway type, suggesting no clear evidence that airway type modified the association between ventilation strategy and delivered inspiratory tidal volume in this cohort.

**Table 2:** Comparison of delivered inspiratory tidal volume between ventilation setting groups using linear mixed-effects model adjusted for potential confounders*.

Variable	Adjusted absolute difference (95% CI)	*p*
Age	0.45 (−0.65, 1.55)	0.426
Male sex	27.16 (−10.08, 64.40)	0.153
Measurement time point, per minute	0.94 (0.60, 1.28)	<0.001
Precomorbidities		
DM	30.55 (−8.32, 69.41)	0.123
HTN	−31.85 (−69.36, 5.66)	0.096
COPD/Asthma	57.46 (−39.41, 154.33)	0.245
CVA	−48.65 (−126.45, 29.16)	0.22
Witnessed arrest	14.80 (−21.86, 51.46)	0.429
Bystander CPR	44.32 (−5.56, 94.19)	0.082
Epinephrine	6.44 (−40.33, 53.21)	0.787
Defibrillation	−4.54 (−44.95, 35.86)	0.826
Advanced airway		
LMA	Reference	
ETT	−57.30 (−98.20, −16.39)	0.006
Ventilation setting groups		
Group 1	19.29 (−27.47, 66.05)	0.419
Group 2	−47.56 (−88.08, −7.04)	0.021
Group 3	Reference	
*Post-hoc* comparison		
Group 1 vs. Group 2	66.84 (21.27, 112.42)	0.012
Group 1 vs. Group 3	19.29 (−27.47, 66.05)	1
Group 2 vs. Group 3	−47.56 (−88.08, −7.04)	0.064

Group 1: ventilated by ATVs with pressure limit set at 45 cmH2O; Group 2: ventilated by ATVs with pressure limit set at 60 cmH2O; Group 3: ventilated manually by BVDs.

ATV, automatic transport ventilator; BVD, bag-valve device; CI, confidence interval; COPD, chronic obstructive pulmonary disease; CPR, cardiopulmonary resuscitation; CVA, cerebrovascular disease; DM, diabetes mellitus; ETT, endotracheal tube; HTN, hypertension; LMA, laryngeal mask airway.

*Measurement time point was modeled as elapsed time from the start of ventilation feedback monitoring. Patient identifier was included as a random intercept. *P*-values for *post-hoc* comparisons were adjusted using Bonferroni correction.

### Association between advanced airway type, ventilation setting, tidal volume, and patient outcomes

Table 3 presents the results of the multivariate logistic regression analysis for factors associated with patient outcomes. Factors associated with a higher risk of prehospital ROSC included older age (adjusted odds ratio [aOR]: 1.04, 95% CI: 1.00–1.08; *p* = 0.035) and ETT insertion (aOR: 22.61, 95% CI: 2.44–209.11; *p* = 0.006). Factors associated with higher odds of any ROSC included witnessed arrest (aOR: 2.48, 95% CI: 1.14–5.41; *p* = 0.022) and prehospital ETT insertion (aOR: 2.61, 95% CI: 1.01–6.71; *p* = 0.047). No significant association was observed among ventilation setting, delivered inspiratory tidal volume, and patient outcomes.

**Table 3:** Factors associated with prehospital ROSC and any ROSC during ED resuscitation.

Variable	Prehospital ROSC		ROSC	
	Adjusted OR (95% CI)	*p*	Adjusted OR (95% CI)	*p*
Age	1.04 (1.00–1.08)	0.035	1.02 (0.99–1.04)	0.220
Male	0.75 (0.25–2.22)	0.605	1.45 (0.63–3.34)	0.377
Precomorbidities				
DM	0.80 (0.25–2.62)	0.717	1.40 (0.61–3.21)	0.430
HTN	0.91 (0.31–2.66)	0.856	1.14 (0.51–2.55)	0.742
COPD/Asthma	NA*		0.40 (0.04–4.00)	0.435
CVA	NA*		NA*	
Witness	2.00 (0.71–5.65)	0.193	2.48 (1.14–5.41)	0.022
Bystander CPR	0.72 (0.15–3.42)	0.678	0.93 (0.31–2.83)	0.904
Epinephrine	1.19 (0.19–7.39)	0.854	0.83 (0.30–2.26)	0.709
Defibrillation	1.24 (0.39–3.97)	0.719	1.53 (0.66–3.58)	0.322
Advanced airway				
LMA	Reference		Reference	
ETT	22.61 (2.44–209.11)	0.006	2.61 (1.01–6.71)	0.047
Ventilation setting				
Group 1	1.15 (0.32–4.13)	0.830	1.01 (0.37–2.75)	0.988
Group 2	0.47 (0.13–1.66)	0.239	0.56 (0.22–1.39)	0.211
Group 3	Reference		Reference	
Tidal volume delivery performance				
Median delivered inspiratory tidal volume (mL)	1.00 (1.00–1.01)	0.655	1.00 (1.00–1.00)	0.991
Maximum delivered inspiratory tidal volume (mL)	1.00 (1.00–1.00)	0.461	1.00 (1.00–1.00)	0.844
Percentage of volume between 500 and 600 ml (%)	1.01 (0.97–1.06)	0.636	1.02 (0.99–1.05)	0.297

Group 1: ventilated by ATVs with pressure limit set at 45 cmH2O.

Group 2: ventilated by ATVs with pressure limit set at 60 cmH2O.

Group 3: ventilated manually by Ambu bags.

CI, confidence interval; COPD, chronic obstructive pulmonary disease; CPR, cardiopulmonary resuscitation; CVA, cerebrovascular disease; DM, diabetes mellitus; ED, emergency department; ETT, endotracheal tube; HTN, hypertension; LMA, laryngeal mask airway; OR, odds ratio; ROSC, return of spontaneous circulation.

## Discussion

In this study, we compared the tidal volume delivery performance between BVDs and ATVs with different pressure limits in patients with OHCA monitored using a ventilation feedback device. Despite slight differences in delivered inspiratory tidal volumes measured across the groups, no statistically significant association with patient outcomes was observed. These findings should be interpreted as exploratory and hypothesis-generating rather than as evidence of equivalence or interchangeability between ATVs and BVDs. This study also does not support the hypothesis that increasing the pressure limit threshold improves delivered inspiratory tidal volume. To the best of our knowledge, previous studies have rarely presented the quality and efficacy of various ventilation methods and settings for patients with OHCA during chest compressions, particularly in prehospital settings. Therefore, our study may provide preliminary evidence for future studies on ventilation during CPR.

This study did not support the hypothesis that increasing the pressure limit of ATVs could enhance the delivered inspiratory tidal volume during CPR. The finding that the 45 cmH2O group had higher delivered inspiratory tidal volume than the 60 cmH2O group appears counterintuitive under conventional respiratory physiology and should not be interpreted as evidence that a lower pressure limit is physiologically superior. Several factors may explain this apparently paradoxical result. First, the comparison between the 45 and 60 cmH2O settings was based on a before-and-after design rather than random allocation. Therefore, the observed difference may reflect temporal effects or residual confounding rather than the isolated effect of pressure limit. Patient characteristics, EMS team practice, provider experience, airway management conditions, device attachment timing, and operational circumstances may have differed between the two periods. Second, the pressure limit of the ATV represents an upper safety threshold for airway pressure, not a target pressure and not a guarantee of increased volume delivery. During CPR, delivered ventilation is influenced by dynamic respiratory mechanics, including chest compression-related changes in intrathoracic pressure, reduced respiratory compliance, airway resistance, airway obstruction, breath timing relative to chest compressions, and potential airway leakage. Under these conditions, increasing the pressure limit may permit higher transient airway pressure without necessarily increasing effective lung inflation or device-derived delivered inspiratory tidal volume. Third, airway-related factors may have contributed. In our cohort, the proportion of patients receiving LMA was numerically higher in the 60 cmH2O group than in the 45 cmH2O group. Although advanced airway type was adjusted for in the LMM and additional stratified analyses were performed, residual differences in airway seal, leakage, airway position, and airway resistance may not have been fully captured by a simple ETT/LMA covariate. Fourth, measurement-related factors should also be considered. The Zoll X Series Real BVM Help system calculates delivered inspiratory tidal volume from inspiratory flow measured at the airway. During ongoing CPR, flow-sensor measurements may be affected by chest compression-related airflow, rapid changes in intrathoracic pressure, airway leak, secretions, device movement, or other artifacts. Therefore, extreme values in Figure 2 may represent transient measurements or artifacts rather than stable effective alveolar ventilation. In a prospective study in prehospital settings, Yang et al. compared the use of different bag sizes for ventilation in patients with OHCA; however, they failed to demonstrate that larger bag sizes provided higher tidal volumes (8). The authors suggest that simply changing the ventilation equipment does not result in measurable physiological differences, considering the significantly altered respiratory mechanics during chest compressions (8). Delivering ventilation between intervals of compressions, such as that practiced with a 30:2 chest compression-to-ventilation ratio, may minimize the significant effect of chest compressions on ventilation. However, this approach increases the duration of chest compressions interruptions. Therefore, current CPR guidelines recommend asynchronous ventilation at a rate of 1 breath every 5 s after advanced airway placement. In contrast, previous studies have proposed the chest compression-synchronized mode, which involves the delivery of a short positive-pressure ventilation precisely at the start of each chest compression. This ventilation mode is suggested to increase PaO2 by leveraging the principle of an “artificial cough resuscitation effort” (12, 13). The relationship between chest compressions, ventilation, and their actual effects on the physiology and outcomes of patients experiencing cardiac arrest remains an unresolved mystery.

In this exploratory cohort, no significant difference in delivered inspiratory tidal volume or patient outcomes was observed between ventilation using ATVs and BVDs. Therefore, ATVs may serve as a practical alternative to manual ventilation using BVDs in this prehospital OHCA cohort, particularly because ATVs can reduce the manpower required for ventilation during prehospital care and provide a consistent ventilation rate. However, this study was not designed to establish formal feasibility, equivalence, or interchangeability between ATV and BVD ventilation. Formal feasibility outcomes, including failed ATV connection, device malfunction, protocol deviation, and non-feasible ATV use, were not prospectively collected. Therefore, these findings require validation through prospective studies with standardized protocols, concurrent comparison groups, comprehensive respiratory monitoring, and adequate power for clinical outcomes.

This study has several limitations that should be acknowledged. First, the retrospective nature of this study introduces potential biases related to data collection and selection, which may affect the generalizability of the findings. For example, the choice of using 45 cmH2O or 60 cmH2O as the pressure limit criteria was based on a sequential before-and-after approach, and whether the patient received ATV or BVD ventilation depended on whether the EMS team was equipped with or applied ATV devices. These factors could introduce temporal confounding, selection bias, confounding by indication, and unmeasured operational confounding related to EMS team characteristics, provider experience, equipment availability, and resuscitation context. Second, the current study relied on data from a single EMS system in Hsinchu County, Taiwan, which may not be representative of other regions with different prehospital care protocols and patient demographics. Third, the sample size was relatively small, and the number of clinical outcome events was very limited, particularly for survival to discharge and good functional outcome. Therefore, the outcome analyses were underpowered and at risk of model overfitting and unstable estimates with wide confidence intervals. The absence of statistically significant outcome differences should not be interpreted as proof of no effect. Fourth, the assessment of ventilation was limited to inspiratory flow-derived delivered inspiratory tidal volume. This device-derived process measure does not directly measure expiratory tidal volume, leakage, alveolar ventilation, dead-space ventilation, oxygenation, carbon dioxide clearance, or gas exchange. Other critical parameters of ventilation, such as airway pressure, expiratory tidal volume, EtCO2, oxygenation parameters, arterial blood gas analysis, and measures of leakage, were not included. Therefore, our data cannot comprehensively assess effective ventilation during CPR. Finally, this study did not account for potential confounding factors, such as the skill level of EMS providers or variations in CPR quality, which could have influenced the outcomes. Future studies with larger multicenter cohorts, prospective randomized designs, standardized ventilation protocols, concurrent comparison groups, and comprehensive respiratory mechanics monitoring are necessary to validate the findings of this study and more comprehensively explore the effects of different ventilation strategies on patient outcomes.

In conclusion, our study found that ATVs demonstrated comparable delivered inspiratory tidal volume to BVD ventilation in this exploratory retrospective cohort of prehospital OHCA patients, but this finding should not be interpreted as evidence of equivalence or interchangeability. Increasing the pressure limit of ATVs from 45 to 60 cmH2O did not increase delivered inspiratory tidal volume. The optimal pressure settings of ATVs, formal feasibility and implementation outcomes, and their effects on clinical outcomes remain unclear. Further prospective studies with standardized protocols, concurrent comparison groups, comprehensive respiratory monitoring, and adequate power for clinical outcomes are necessary to validate these findings.

## Data Availability

The raw data supporting the conclusions of this article will be made available by the authors, without undue reservation.
