# Supplementary material for: Comparison of prehospital tidal volume delivery performance between automated transport ventilators and bag-valve devices in out-of-hospital cardiac arrest patients: does the pressure limit matter?
Source: Front Med (Lausanne). 2026 May 25;13:1826906. doi: 10.3389/fmed.2026.1826906 (PMC13243249; doi:10.3389/fmed.2026.1826906)
Supplement: Supplementary file 1 [file Table_1.DOCX]

| **Supplementary Table 1.** Comparison of ventilation efficacy between groups and different advanced airway type | | | | | | | |
| --- | --- | --- | --- | --- | --- | --- | --- |
|  |  | Total (n = 178) | Group 1 (n = 41) | Group 2 (n = 80) | Group 3 (n = 57) | *p* | P* |
| Median tidal volume (mL) | Total | 333.0 (229.0) | 403.0 (127.0) | 275.0 (244.8) | 363.0 (228.8) | 0.016 | 0.027 |
|  | LMA | 381.0 (196.5) | 438.5 (519.0) | 310.5 (220.3) | 401.0 (147.5) | 0.035 |  |
|  | ETT | 311.0 (250.5) | 388.5 (219.3) | 255.5 (235.0) | 319.0 (236.5) | 0.041 |  |
| Max tidal volume (mL) | Total | 577.5 (225.0) | 534.0 (163.0) | 567.5 (238.0) | 644.0 (246.0) | 0.024 | 0.155 |
|  | LMA | 601.0 (249.0) | 619.0 (297.0) | 588.5 (228.0) | 656.0 (252.0) | 0.320 |  |
|  | ETT | 549.0 (225.0) | 518.0 (144.0) | 516.0 (244.0) | 641.5 (249.0) | 0.018 |  |
| Percentage of volume between 500-600 ml (%) | Total | 3.5 (16.0) | 3.1 (20.1) | 2.0 (12.0) | 5.7 (16.0) | 0.372 | 0.034 |
|  | LMA | 6.6 (18.2) | 11.7 (26.3) | 3.6 (17.7) | 8.0 (23.1) | 0.448 |  |
|  | ETT | 2.5 (12.1) | 1.5 (20.9) | 0.9 (9.2) | 3.7 (13.5) | 0.445 |  |
| Group 1: ventilated by ATVs with pressure limit set at 45 cmH2O  Group 2: ventilated by ATVs with pressure limit set at 60 cmH2O  Group 3: ventilated manually by Ambu bags  P*: Comparison between LMA group and ETT group ETT=endotracheal tube, LMA=laryngeal mask airway | | | | | | | |

| **Supplementary Table 2.** Stratified analysis of delivered inspiratory tidal volume by advanced airway type* | | | |
| --- | --- | --- | --- |
| **Advanced airway type** | **Comparison** | **Adjusted Absolute Difference (95% CI)** | ***p*** |
| LMA | Group 1 vs Group 2 | 56.75 (-36.00, 149.51) | 0.691 |
| LMA | Group 1 vs Group 3 | 10.28 (-98.79, 119.34) | 1.000 |
| LMA | Group 2 vs Group 3 | -46.48 (-112.96, 20.00) | 0.512 |
| ETT | Group 1 vs Group 2 | 61.90 (4.12, 119.68) | 0.107 |
| ETT | Group 1 vs Group 3 | 9.10 (-48.34, 66.54) | 1.000 |
| ETT | Group 2 vs Group 3 | -52.80 (-106.59, 0.98) | 0.163 |
| Group 1: ventilated by ATVs with pressure limit set at 45 cmH2O; Group 2: ventilated by ATVs with pressure limit set at 60 cmH2O; Group 3: ventilated manually by BVDs. *Adjusted differences were estimated using linear mixed-effects models stratified by advanced airway type. Patient identifier was included as a random intercept, and measurement time point was included as a fixed effect. Models were adjusted for age, sex, pre-existing comorbidities, witnessed arrest, bystander CPR, epinephrine administration, and defibrillation. COPD/asthma was excluded from the LMA subgroup model because all patients in this subgroup had no recorded COPD/asthma. P-values were adjusted using Bonferroni correction for pairwise comparisons. ATV = automatic transport ventilator; BVD = bag-valve device; CI = confidence interval; ETT = endotracheal tube; LMA = laryngeal mask airway. | | | |
